# Defining the optimal cut-off values for liver enzymes in diagnosing blunt liver injury

**DOI:** 10.1186/s13104-016-1863-3

**Published:** 2016-01-25

**Authors:** Tomohide Koyama, Hirohisa Hamada, Masamichi Nishida, Paal A. Naess, Christine Gaarder, Tetsuya Sakamoto

**Affiliations:** Department of Emergency Medicine, Teikyo University Hospital, 2-11-1 Kaga, Itabashi, Tokyo, Japan; Department of Emergency Medicine, Toranomon Hospital, Tokyo, Japan; Department of Traumatology, Oslo University Hospital-Ulleval, Oslo, Norway

**Keywords:** Blunt liver trauma, Liver transaminase, CE-MDCT, ROC curve analysis, Youden index

## Abstract

**Background:**

Patients with blunt trauma to the liver have elevated levels of liver enzymes within a short time post injury, potentially useful in screening patients for computed tomography (CT). This study was performed to define the optimal cut-off values for serum aspartate aminotransferase (AST) and alanine aminotransferase (ALT) in patients with blunt liver injury diagnosed with contrast enhanced multi detector-row CT (CE-MDCT).

**Methods:**

All patients admitted from May 2006 to July 2013 to Teikyo University Hospital Trauma and Critical Care Center, and who underwent abdominal CE-MDCT within 3 h after blunt trauma, were retrospectively enrolled. Using receiver operating characteristic (ROC) curve analysis, the optimal cut-off values for AST and ALT were defined, and sensitivity and specificity were calculated.

**Results:**

Of a total of 676 blunt trauma patients 64 patients were diagnosed with liver injury (Group LI+) and 612 patients without liver injury (Group LI−). Group LI+ and LI− were comparable for age, Revised Trauma Score, and Probability of survival. The groups differed in Injury Severity Score [median 21 (interquartile range 9–33) vs. 17 (9–26) (p < 0.01)]. Group LI+ had higher AST than LI− [276 (48–503) vs. 44 (16–73); p < 0.001] and higher ALT [240 (92–388) vs. 32 (16–49); p < 0.001]. Using ROC curve analysis, the optimal cut-off values for AST and ALT were set at 109 U/l and 97 U/l, respectively. Based on these values, AST ≥ 109 U/l had a sensitivity of 81 %, a specificity of 82 %, a positive predictive value of 32 %, and a negative predictive value of 98 %. The corresponding values for ALT ≥ 97 U/l were 78, 88, 41 and 98 %, respectively, and for the combination of AST ≥ 109 U/l and/or ALT ≥ 97 U/l were 84, 81, 32, 98 %, respectively.

**Conclusions:**

We have identified AST ≥ 109 U/l and ALT ≥ 97 U/l as optimal cut-off values in predicting the presence of liver injury, potentially useful as a screening tool for CT scan in patients otherwise eligible for observation only or as a transfer criterion to a facility with CT scan capability.

## Background

The liver is one of the most commonly injured abdominal organs and is reported in approximately 5 % of all trauma patients [1, 2]. Since computed tomography (CT) was introduced in trauma evaluation in the early 1980s [3], patients with a history of significant trauma who are hemodynamically normal(ized) will undergo CT if available. Although CT has become the “gold standard” for detecting injuries to the intraabdominal solid organs, CT is not always present in every institution worldwide, even in high-income countries such as Japan. Additionally, there is evidence demonstrating that CT scanning carries a risk of causing malignancies and thus should be avoided when possible [4].

Ultrasound has significant limitations as a diagnostic tool since the overall sensitivity is as low as 72 % for detecting blunt liver injury based on detection of free fluid, parenchymal injury or both [5].

On that background the serum biomarkers such as serum aspartate aminotransferase (AST) and alanine aminotransferase (ALT) have received attention as markers of liver injury. Several previous studies have tried to define the cut-off value for AST and/or ALT in blunt liver injury [6–15]. However, the results have been conflicting. We infer that the variations might be related to biases such as the population studied, the detection method for liver injury, timing of blood sampling, and statistical analysis method.

The purpose of this study was to establish optimal cut-off values for AST and ALT in patients with blunt liver injury. Such values could be potentially useful to indicate the need for CT scan in patients otherwise eligible for observation only or as a transfer criterion to a facility with CT scan capability.

## Methods

Based on the results from a previous study from our institution published in a Japanese journal [16], all blunt trauma patients admitted to Teikyo University Hospital Trauma and Critical Care Center who underwent initial evaluation with abdominal contrast enhanced (CE) multi detector-row computed tomography (MDCT) within 3 h after injury, were retrospectively enrolled between May 2006 and July 2013. This study was approved by Teikyo University Hospital Ethics Committee.

Admission data collected included the following: age, gender, mechanism of injury, Glasgow Coma Scale (GCS), and Revised Trauma Score (RTS). All patients included in the study were followed throughout their hospital stay. Injury Severity Score (ISS), Probability of survival (Ps), interventions (laparotomy and/or angioembolization (AE)), liver related complications, and mortality were recorded.

The admission values of AST and ALT were measured using LABOSPECT 008 Automatic Analyzer or Clinical Analyzer Model 7600 (both Hitachi High- Technologies Corporation (Corp.), Tokyo, Japan).

Hemodynamically normal patients, on admission or after initial resuscitation, underwent CE-MDCT if at least one of the following criteria was fulfilled: (1) complaint of severe abdominal pain, (2) peritonism, (3) external signs of abdominal injuries, (4) presence of hematuria, melena or hematemesis, (5) abnormal radiographic findings commonly associated with abdominal injuries (intraperitoneal free air, lower rib fracture, pelvic fracture, or lumbar fracture) (6) positive abdominal focused assessment with sonography in trauma (FAST), (7) acute anemia with hemoglobin <10 g/dl, (8) impaired consciousness due to suspected traumatic brain injury.

CT was performed using a 64-slice MDCT scanner (Aquilion 64, TSX-101A/HA, Toshiba Medical Systems Corp., Japan) with intravenous contrast material (Omnipaque 300 injection syringe, Daiichi Sankyo Company (Co.), Limited (Ltd.), Tokyo, Japan or Oypalomin 300 injection syringe, Fuji Pharma Co., Ltd., Toyama, Japan) unless the patient was known to suffer from chronic kidney disease.

The liver injury was defined from CE-MDCT scans based on Organ Injury Scale (OIS, 1994 revision) described by the American Association for the Surgery of Trauma [17]. Attending staff reviewed the CE-MDCT at the following morning conference, and consensus was reached.

Statistical analysis was performed using the IBM SPSS Statistics version 22 for MacOSX [International Business Machines Corp., New York, United States of America (USA)] and the Microsoft Excel for Mac 2011 (Microsoft Corp., Washington, USA). Categorical variables were presented as medians and underwent Chi square test. Continuous variables were presented as median with interquartile range (IQR), and subjected to the Mann–Whitney U test. All p values reported are two-sided, and p values <0.05 were considered to indicate statistical significance.

Receiver operating characteristic (ROC) curve analysis was performed to define the optimal cut-off values for AST and ALT [18]. Two additional analysis methods were used to determine the optimal cut-off values objectively. The first method was ‘The closest to (0, 1) criteria’, in this paper called ‘upper-left (UL) index’, and represents the values at the shortest distance from the upper left corner to the ROC curve. The second was ‘the Youden index’, which describes the maximum vertical distance between the ROC curve and the diagonal or chance line [19]. After determining the optimal cut-off values for AST and ALT with these methods, sensitivity and specificity were calculated.

## Results

**Fig. 1:**
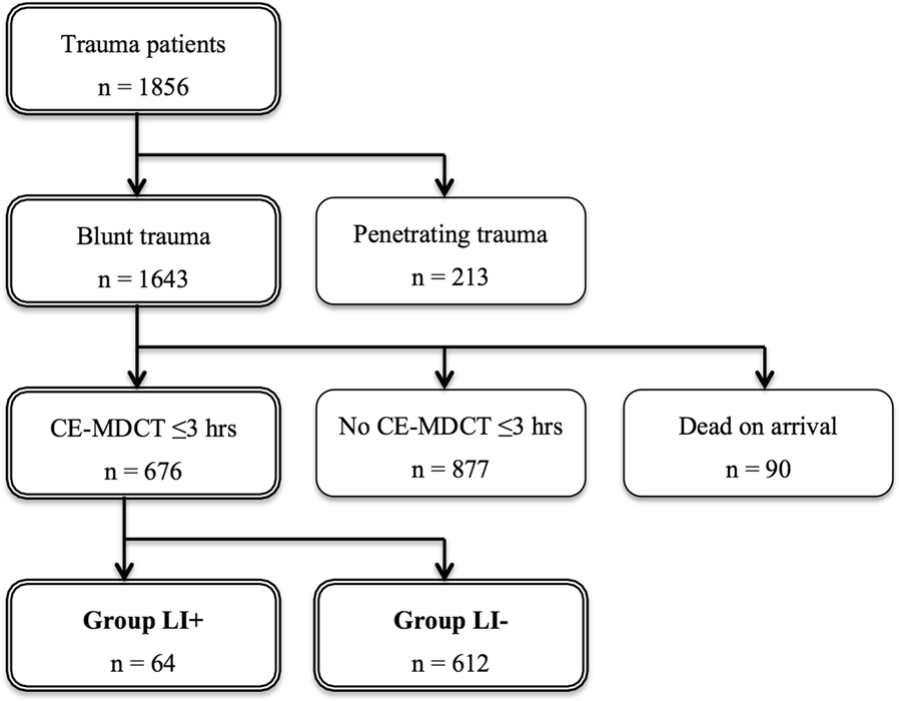
Flowchart of study population. *CE-MDCT* contrast enhanced multi detector-row computed tomography; *LI*+ with liver injury, *LI*− without liver injury

During the study period, 1856 trauma patients were admitted. Of the 1643 patients with blunt trauma, 676 patients underwent abdominal CE-MDCT within 3 h after injury. Based on CE-MDCT scans, 64 patients were diagnosed with liver injury (Group LI+) and 612 patients without liver injury (Group LI−) (Fig. 1).

Group LI+ consisted of nine patients with OIS grade I injuries, 30 patients with grade II injuries, 18 with grade III injuries, 6 with grade IV injuries, and 1 with grade V injury. In Group LI+, 9 (14.1 %) patients had isolated liver injury and 55 (85.9 %) patients had combined injuries. Of the 55 patients with combined injuries, 17 patients had head injury, 12 had facial injury, 45 had chest injury, 21 had other abdominal injuries, 12 had pelvic injury, 18 had spinal injury, and 20 had extremity injury.

In Group LI+, 11 (17.2 %) patients underwent AE and 5 (7.8 %) underwent laparotomy. A total of 5 (7.8 %) patients developed liver related complications. Two patients had biloma, one was treated with percutaneous drainage, and one resolved spontaneously. One patient had bile leakage treated with surgical drainage, one patient had a pseudo-aneurysm treated with AE, and one patient had cholecystitis treated with percutaneous transhepatic gallbladder drainage.

Five (7.8 %) patients in Group LI+ died from massive hemorrhage, none of them liver-related; three patients with pelvic fracture with retroperitoneal hematoma, and two patients with chest injury.

Characteristics of this study population are presented in Table 1. Group LI+ and LI− were comparable for age, RTS, and Ps. The groups differed in ISS [median 21 (IQR 9–33) vs. 17 (9–26); p < 0.01]. Group LI+ had higher AST than LI− [276 (48–503) vs. 44 (16–73); p < 0.001] and higher ALT [240 (92–388) vs. 32 (16–49); p < 0.001].

**Table 1:** Characteristics of the study population

	All (n = 676)	LI+ (n = 64)	LI− (n = 612)	p value
AST	48 (31–106)	276 (48–503)	44 (16–73)	<0.001
ALT	36 (22–69)	240 (92–388)	32 (16–49)	<0.001
ISS	17 (9–26)	21 (9–33)	17 (9–26)	<0.01
GCS	15 (13–15)	15 (14–15)	14 (13–15)	0.67
RTS	7.84 (6.90–7.84)	7.84 (7.37–7.84)	7.84 (7.37–7.84)	0.32
Ps	97.0 (89.5–99.2)	95.7 (88.1–100)	97.4 (92.9–100)	0.19
Age	46 (29–63)	40 (23–57)	46 (29–63)	0.17
Male gender, n (%)	462 (68.3)	36 (56.3)	426 (69.6)	0.03
Mortality, n (%)	58 (8.6)	5 (7.8)	53 (8.7)	0.82

Values are given as median (IQR) where not stated otherwise

*LI*+ with liver injury, *LI*− without liver injury, *AST* aspartate aminotransferase, *ALT* alanine aminotransferase, *ISS* injury severity score, *GCS* Glasgow coma scale, *RTS* revised trauma score, *Ps* probability of survival

**Fig. 2:**
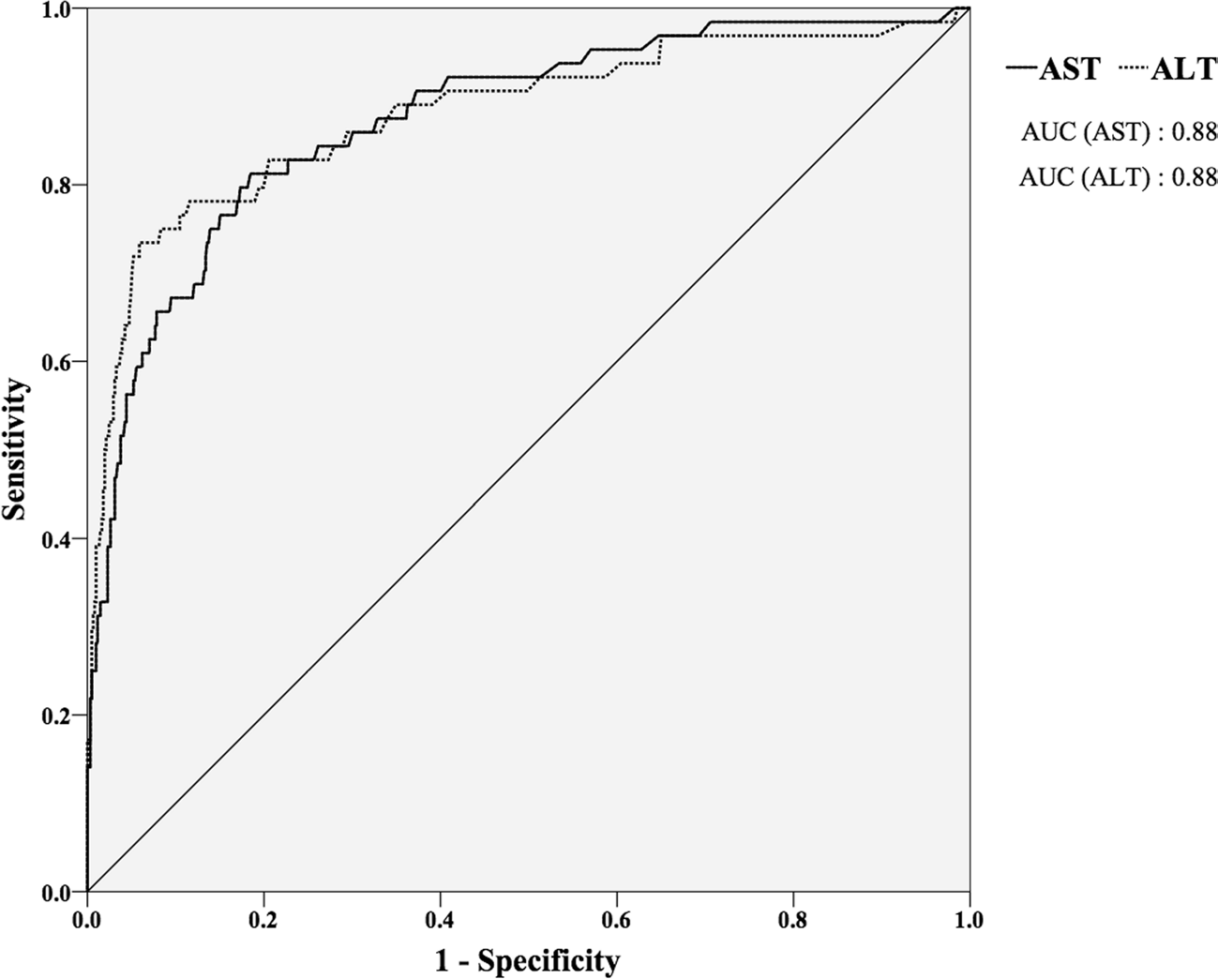
ROC curve of AST and ALT. *AST* aspartate aminotransferase, *ALT* alanine aminotransferase, *ROC* receiver operating characteristic, *AUC* area under the curve

**Fig. 3:**
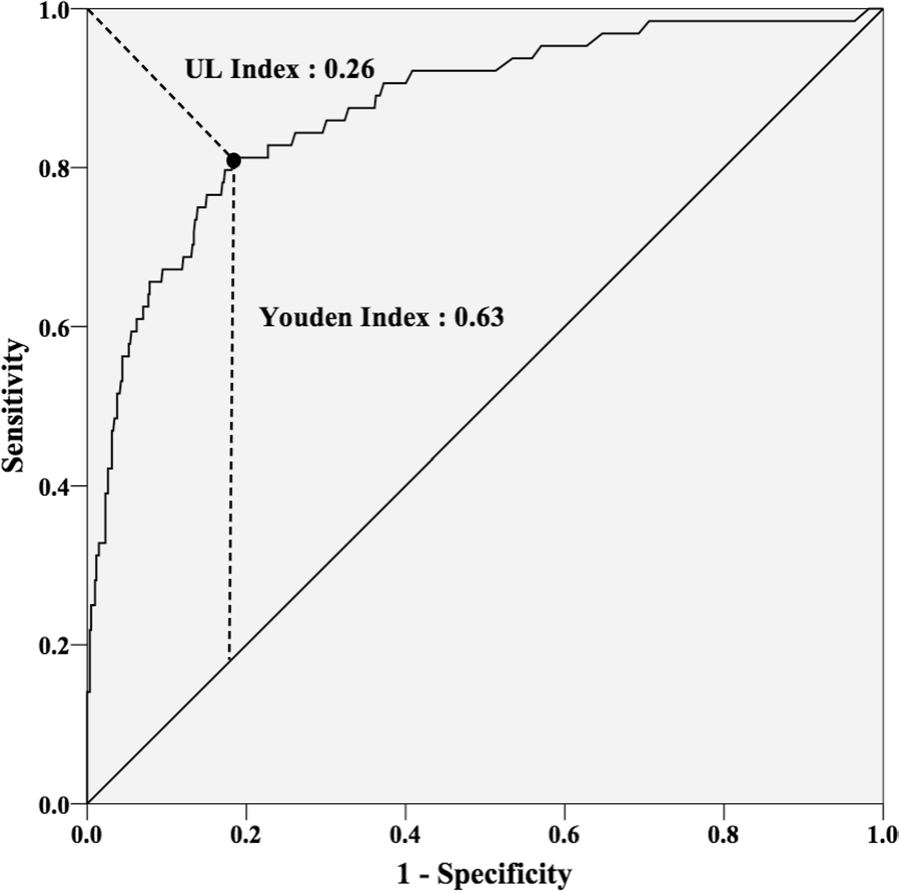
ROC curve of AST with UL index and Youden index. *ROC* receiver operating characteristic, *AST* aspartate aminotransferase, *UL* upper-left

**Fig. 4:**
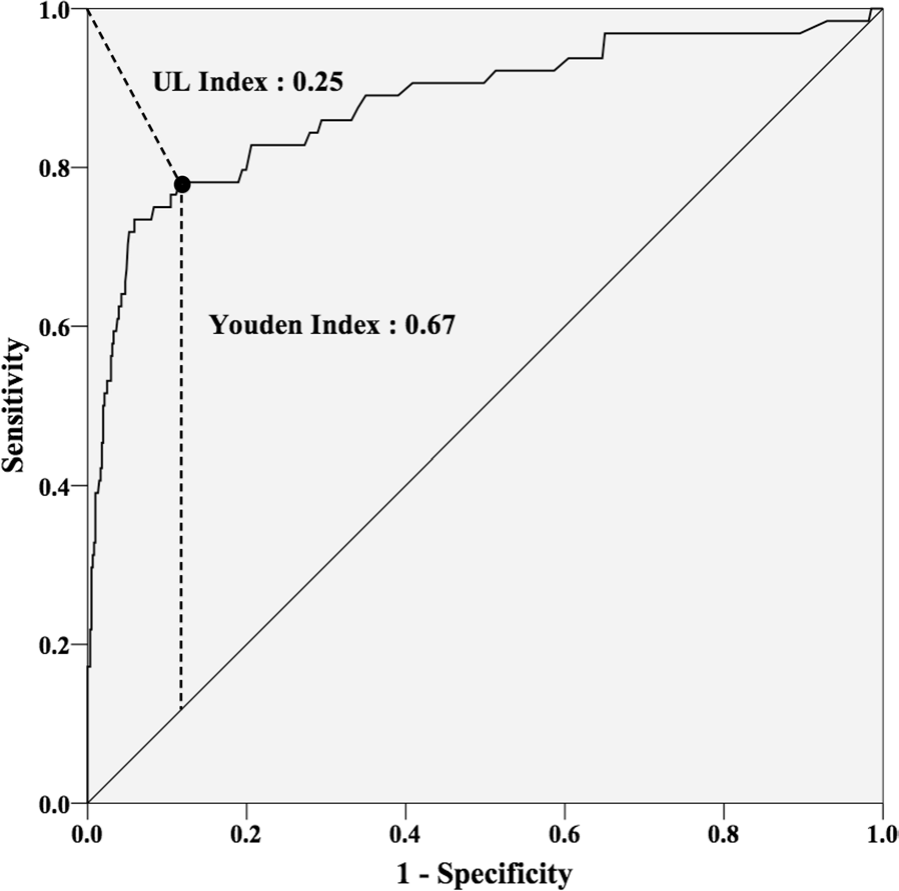
ROC curve of ALT with UL index and Youden index. *ROC* receiver operating characteristic, *ALT* alanine aminotransferase, *UL* upper-left

ROC curve analysis for AST and ALT was performed where the area under ROC curve (AUC) of AST was 0.88 (95 % confidence interval (CI) 0.83–0.92) and of ALT was 0.88 (95 % CI 0.83–0.94) (Fig. 2). With these analyses, the optimal cut-off values for AST was set at 109 U/l (UL index 0.26, Youden index 0.63) (Fig. 3) and ALT were set at 97 U/l (UL index 0.25, Youden index 0.67) (Fig. 4), and the calculated sensitivity and specificity based on these cut-off values are shown in Table 2.

**Table 2:** Results of the optimal cut-off values of AST and ALT

Cut-off values	Sensitivity (%)	Specificity (%)	PPV (%)	NPV (%)
AST ≥ 109	81	82	32	98
ALT ≥ 97	78	88	41	98
AST ≥ 109 and/or ALT ≥ 97	84	81	32	98

*LI*+ with liver injury, *LI*− without liver injury, *AST* aspartate aminotransferase, *ALT* alanine aminotransferase, *PPV* positive predictive value, *NPV* negative predictive value

Ten patients with AST < 109 U/l and ALT < 97 U/l had liver injury diagnosed on CE-MDCT; one OIS grade I injury, eight grade II, and one grade III. None of these ten patients required any interventions for their liver injury, and didn’t suffer any liver related complication or death.

## Discussion

In the present study, blood samples were drawn immediately upon arrival and CT scans performed within 3 h post injury. Based on these strict criteria and the use of UL index and Youden index, we defined AST ≥ 109 U/l and ALT ≥ 97 U/l as the optimal cut-off values in predicting the presence of blunt liver injury.

Shadev et al. [12] used ROC curve analysis to define cut off values for AST and ALT in patients with blunt liver injury verified by ultrasound, diagnostic peritoneal lavage, nuclear scanning, laparotomy or CT-scan. Moreover, 50 % of the patients in the non-liver injury group were identified based on physical examination alone. The method for defining the optimal cut-off values for AST and ALT was not described.

In a study by Tian et al. [13] the cut-off values were set at AST ≥ 113 U/l and ALT ≥ 57 U/l by using ROC curve analysis. To identify the liver injury, CT and laparotomy were used and blood samples were drawn up to 24 h after injury. Moreover, the values of AUC for AST and ALT and the method for defining the optimal cut-off values were not described in their study.

Tan et al. [14] set the cut-off values for AST ≥ 83 U/l and ALT ≥ 64 U/l in a series of 99 patients of whom 55 patients were LI+ defined by CT and laparotomy. In a case–control study, Lee et al. [15] set the cut-off values at AST ≥ 100 U/l and ALT ≥ 80 U/l. They compared 42 LI+ patients and 42 LI− patients based on findings on CT evaluation. Statistical analysis was done to determine whether AST and ALT could predict the liver injury. However, in none of those studies any attempt to define the optimal cut-off values for AST and ALT were performed.

As many as 10 LI+ patients presented with AST < 109 U/l and ALT < 97 U/l. However, they were all treated by observation alone and suffered no complications. Thus, the cut-off values in the present study seem to represent clinically relevant thresholds for further diagnostics, especially in a remote and/or resource limited institution, to decide whether a patient is eligible for observation alone without jeopardizing the patient’s safety or require closer monitoring and further investigations.

In the aforementioned study of Hamada et al. [16] the optimal cut-off values for AST and ALT were defined to be 166 U/l and 130 U/l, respectively. We assume that the lower cut-off values for AST and ALT in the present study can be attributed to the higher detection capability of the MDCT compared to the one achievable in the single detector-row computed tomography [20–22] as used in the previous study.

There are some limitations to this study in addition to its retrospective nature. The number of LI+ patients was relatively small. Additional studies are required to verify whether the optimal cut-off values defined in a single urban center in Asia are applicable worldwide.

## Conclusions

In conclusion, we have identified AST ≥ 109 U/l and ALT ≥ 97 U/l as optimal cut-off values for predicting the presence of liver injury in blunt trauma, potentially useful as a screening tool for CT scan in patients otherwise eligible for observation only or as a transfer criterion to a facility with CT scan capability.
